# Longitudinal association of perfluorooctanoic acid (PFOA) and perfluorooctanesulfonic acid (PFOS) exposure with lipid traits, in a healthy unselected population

**DOI:** 10.1038/s41370-025-00773-3

**Published:** 2025-04-24

**Authors:** Yasrab N. Raza, Julia S. El-Sayed Moustafa, Xinyuan Zhang, Dongmeng Wang, Max Tomlinson, Mario Falchi, Cristina Menni, Ruth C. E. Bowyer, Claire J. Steves, Kerrin S. Small

**Affiliations:** 1 Department of Twin Research and Genetic Epidemiology, King’s College London, London, UK; 2 Department of Pathophysiology and Transplantation, Università Degli Studi di Milano, Via Francesco Sforza, 35, 20122 Milan, Italy; 3 Fondazione IRCCS Cà Granda Ospedale Maggiore Policlinico, Angelo Bianchi Bonomi Hemophilia and Thrombosis Center, 20122 Milan, Italy

**Keywords:** Perfluorinated chemicals, Epidemiology, PFAS, Health studies

## Abstract

**Background:**

Perfluorooctanoic acid (PFOA) and Perfluorooctanesulfonic Acid (PFOS) are synthetic substances with long half-lives. Their presence is widespread and pervasive, and they are noted for their environmental persistence. Research has shown these chemicals to be associated with dyslipidaemia, although few studies have considered the long-term associations in the general population.

**Objectives:**

The aim of this study was to consider the longitudinal and cross-sectional associations with lipid phenotypes.

**Methods:**

We investigated the association of these chemicals with total cholesterol (TC), low-density lipoprotein (LDL), high-density lipoprotein (HDL), triglycerides (TG), and the total cholesterol: high-density lipoprotein ratio (TC:HDL), in a healthy unselected British population of twins (*n* = 2069), measured at three timepoints between 1996 and 2014.

**Results:**

Serum levels of PFOA and PFOS decreased over time during this period. We demonstrate longitudinal associations across serum levels of both PFOA and PFOS, finding positive associations with TC (PFOA:β = 0.51, *p *= 1.9e−07; PFOS:β = 0.24, *p *= 3.8e−05) and LDL (PFOA:β = 0.61, *p *= 1.7e−11; PFOS:β = 0.42, *p *= 1.6e−14), and consistent negative associations with HDL and PFOA (β = −0.12, *p *= 0.003) and PFOS (β = −0.25, *p *= <2e−16). We also observe cross-sectional associations of PFAS with lipids across all three timepoints.

**Impact:**

- PFAS remain persistent in the environment, despite regulations, due to their structural properties, leaving humans open to exposure. There is less understanding of how chronic low exposure to these chemicals, particularly within an unselected population, may impact health outcomes. This study reports the longitudinal associations of PFOA and PFOS over an 18-year window with 5 lipid phenotypes, highlighting that despite falling serum levels, PFAS exposure may lead to hyperlipidaemia. We further investigate the cross-sectional associations across three timepoints to understand time-dependent effects, demonstrating associations persist. This work aids our understanding on the long-term effect of chronic PFAS exposure.

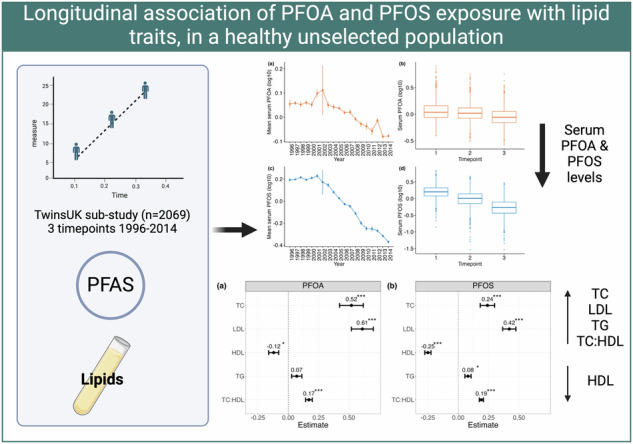

## Introduction

PFOA (perfluorooctanoic acid) and PFOS (perfluorooctanesulfonic acid) are synthetic chemicals, categorised under per- and polyfluoroalkyl substances (PFAS) [1], once extensively used for industrial purposes and consumer products [2]. Due to their structural properties, PFAS are resistant to degradation and continue to persist in the environment long after their production [3, 4]. This persistence and widespread use has resulted in ubiquitous global exposure [5, 6], with human exposure occurring primarily through food, water, air, dust, and consumer products [7]. Additionally, they possess long half-lives, with estimates ranging from 2.7 to 3.8 years for PFOA and 3.4 to 5.4 years for PFOS [8, 9]. These characteristics, and their association with adverse health effects has led to their classification as persistent organic pollutants (POPs), since 2009 (PFOS) and 2020 (PFOA), respectively, resulting in stringent regulations in the UK [10].

Studies on the health effects of PFOA and PFOS have been conducted in a range of populations with differing exposure profiles. For instance, numerous studies have been conducted in cohorts highly exposed via contaminated drinking water [11–13], or occupationally exposed [14–17]. Fewer studies have reported on the effects of PFAS in unselected, general populations [18, 19], with one study in a British population (The Avon Longitudinal Study of Parents and Children), which studied the in-utero effects of PFAS exposure [20].

Several cross-sectional studies have explored the relationship between PFOA and PFOS and their association with serum lipids and hypercholesterolaemia [21–32]. Most studies have shown a positive association between PFOA and PFOS with total cholesterol (TC) and low-density lipoproteins (LDL) [18, 21, 23, 24, 27–29, 31, 33–35]. The relationship with high-density lipoprotein (HDL) and triglycerides (TG) is less certain, with both positive and negative associations reported, and some studies finding no statistically significant associations [17, 21, 23, 31, 36–39].

Several studies have reported the longitudinal associations between PFAS and lipids [25, 29, 36, 40–45]. Of these studies, 5 were occupationally exposed cohorts or communities exposed via contaminated drinking water; 1 was a diabetic case-control cohort; 2 only had baseline levels of PFOA and PFOS measured; and 1 study was reported within the population of Upsala, Sweden. Most of these longitudinal studies continued to support an association between PFOA or PFOS with increased TC or LDL [25, 29, 40–42, 44], although two studies did not find any significant associations [36, 43]. Whilst one study found an inverse association with TG [29], others found PFOA or PFOS to be associated with increased TG or HDL [43–45], whilst two studies did not find any significant associations [40, 41].

While cross-sectional associations between PFOA and PFOS with lipid traits have are generally been well established, the longitudinal effect of these chemicals with lipids is less well studied (9 longitudinal studies, in comparison to the 18 cross-sectional studies referenced here and further 29 highlighted in a recent meta-analysis [46]). Cross-sectional studies are limited since they only consider one point in time and are liable to reverse causation (where the outcome itself may lead to higher serum levels of PFAS [47]). The few longitudinal studies conducted have mainly been in highly exposed populations or with limited follow-up – 5 of the longitudinal studies were conducted in populations who had been occupationally exposed or known exposure via contaminated drinking water, in comparison to and only 1 study in a general population cohort in Sweden.

The purpose of this study is therefore to understand both cross-sectional and longitudinal associations between PFOA and PFOS with lipid traits over an 18-year longitudinal window in a healthy, unselected British population to understand the long-term effect of these PFAS exposure, particularly given their persistence in the environment.

## Methods

### Study population

TwinsUK is an adult twin registry of over 15,000 volunteers with extensive phenotype and genotype data [48]. Data has been collected over the last 30 years, including detailed clinical and biochemical measures, longitudinal data for several phenotypes and annual questionnaire data available for clinical outcomes, including medication use. The TwinsUK cohort has a similar distribution of traits and prevalence of disease, comparable to the age-matched general population [49]. Lipid phenotypes were collected as part of routine visits within TwinsUK, whilst metabolite profiling collection of PFOA/PFOS was conducted separately as part of a sub-study within TwinsUK, which collected three repeated measures of metabolomic data over an 18-year window in 2069 individuals.

### Metabolomic data profiling

PFAS were measured longitudinally in 2069 individuals between 1996 and 2014, where three measures of PFAS were collected equating to 3 timepoints. The three timepoints of serum PFOA/PFOS measures were determined by order of visit, where the first visit was considered the first measurement, the second visit the second measurement and the third visit the third measurement. The longitudinal samples were not collected in fixed sweeps. Instead, samples were collected in waves, during rolling visits to the department. Participants are invited for routine visits on a rolling basis, whilst the PFOA/PFOS measures were collected as part of a sub-study. To reduce excess visits, where possible, data was collected during these routine clinic visits, leading to differences in time between visits. This results in some overlap between timepoints due to varying collection dates across individuals (Supplementary Fig. S1b). There is a mean of 13.2 years in sample collection between the first and last timepoint, with 7.1 years between timepoints 1 and 2 and 6.1 years between timepoints 2 and 3.

PFOA and PFOS levels were captured using the Metabolon panel. Although Metabolon quantification provides relative values, as opposed to absolute measures, measurement of PFAS using the Metabolon platform has been shown to correlate strongly with total serum measures of PFOA and PFOS [50], including previous large-scale work which has shown agreement of 97–98% between targeted methods and non-targeted methods [51].

Metabolomic profiling has been previously described [52]. Briefly, metabolites were measured in serum for 2069 subjects at three timepoints using non-targeted ultra-high performance liquid chromatography tandem mass spectrometry (UPLC-MS/MS) by Metabolon Inc. (Morrisville, USA). Prior to injection into the UPLC-MS/MS serum samples underwent comprehensive processing and quality control. This included methanol extraction, for protein precipitation, dividing the sample extracts into multiple fractions for analysis under different conditions, organic solvent removal and sample reconstitution to solvent compatible with each method. Several controls were also analysed alongside the samples. Pooled serum samples were analysed as technical replicates to assess instrumental variability, with relative standard deviation values for PFOA and PFOS calculated to confirm consistency. Raw data was extracted, peak-identified and QC processed using Metabolon software. Metabolites only detected in fewer than 20% of samples were excluded. Metabolites detected in more than 20% of samples were considered common. Common metabolites were median scaled by day of measurement to a median value of 1. Missing data was imputed using nonparametric missing value imputation using random forest, with the R package missForest [53]. Metabolites have been log10 normalised. A detailed description of the metabolomic profiling is described within the supplemental methods.

### Lipid phenotypes

The following lipids were collected in TwinsUK and measured in mmol/L using a colorimetric assay; Total Cholesterol (TC), High-Density Lipoprotein (HDL), Low-Density Lipoprotein (LDL), Triglycerides (TG). The TC:HDL ratio was calculated dividing TC by HDL. A higher ratio is associated with poorer cholesterol outcomes and this phenotype was used to help interpret the effect of PFOA and PFOS on overall lipid outcomes. Participants are invited to routine clinical visits on a rolling basis every few years during which lipid phenotypes were collected. The metabolite (PFOA/PFOS) samples were collected separately as part of a sub-study. To integrate these data, the lipid measures were matched to the metabolite measures within a 1.5 year window by the date of sample collection. PFOA/PFOS measures were only retained if a corresponding lipid phenotype was measured within this timeframe. 75% of metabolite samples had a matching lipid phenotype measured within this timeframe. Most of these samples (85%, *n* = 3940) were collected on the same day and a further 532 samples were matched to within 1 month (Supplementary Fig. S1a).

### Medication usage

Medication status was determined using questionnaire data, as previously described [54]. Individuals were presumed to be taking lipid medication if they reported taking medication any time prior to the PFAS measurement date or within one year of this date. Individuals taking lipid lowering medication were removed from the analyses.

### Statistical model & analysis

Linear mixed effects model were employed to assess the relationship between the lipid phenotype with PFOA and PFOS, adjusting for age, sex, and BMI as fixed covariates, and with family relatedness and technical covariates fitted as a random intercept. For the longitudinal model, a random slope was also fitted to account for longitudinal variation of PFOA/PFOS between individuals, and an ID variable was included to account for the repeated measures of individuals. This correlates to the following model: lipid ~PFA + age + BMI + sex + (1 + PFA | ID) + (1 | ID) + family relatedness + technical covariates. The technical covariate encompasses the sample processing plate, relevant to the processing of PFOA/PFOS samples, to account for batch effects and technical variability. Family relatedness encompasses random effects for twin structure and family relatedness to account for the variance and relatedness of the samples. This includes a random effect for family ID, which accounts for family relatedness between twin pairs and zygosity which accounts for monozygotic (MZ) twins being genetically more similar in comparison to dizygotic (DZ) twins. BMI, TG and TC:HDL were natural log transformed in the models. All other variables were normally distributed. All analyses were conducted in R (version 4.2.2) using the lmerTest [55] package in R Studio (version 2023.03.0 + 386). A Bonferroni correction was applied, with a significance threshold of *P *< 0.005 (5 tests against 2 variables 0.05/10).

## Results

### Study population characteristics

**Fig. 1:**
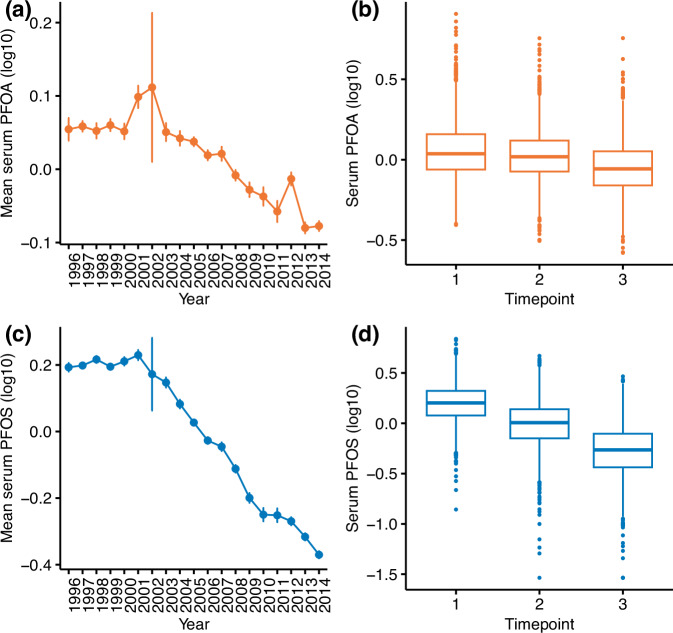
Serum PFOA and PFOS levels across time. Mean serum PFOA (**a**) and PFOS (**c**) levels (with standard errors) at each year between 1996 and 2014. Large standard errors in 2002 are noted due to the very low sample size in this year (*n* = 6). Serum PFOA (**b**) and PFOS (**d**) levels across three timepoints where repeated measured were taken for individuals.

PFAS were measured longitudinally between 1996 and 2014 in 2069 individuals in total, with 3 repeated measures taken during this period. Overall, a decline in serum PFOA and PFOS levels during the 18-year collection period was observed (Fig. 1a, c). We further observe a decline in the median serum PFOA and PFOS levels across each of the three timepoints (Fig. 1b, d). As these PFAS were measured using the Metabolon panel, the values presented are relative and not absolute. Descriptive statistics for the study population, once data was matched to lipid phenotypes, are summarised in Table 1. Due to the removal of individuals taking lipid-lowering medication, and the availability of matched lipid measures, the sample size is reduced and varies across each timepoint. It is an ageing cohort, consisting largely of females and a higher proportion of monozygotic twins. Mean age at the three timepoints was 51, 58 and 64 years, respectively. A modest increase TC and HDL is shown over time, and a decrease in TG and TC:HDL is decreasing across timepoints. LDL levels fluctuates, with an overall decrease between timepoint 1 and timepoint 3.

**Table 1:** Summary statistics by timepoint.

Variable mean (± sd)	Timepoint 1 (*n* = 1145)	Timepoint 2 (*n* = 1601)	Timepoint 3 (*n* = 1269)	*p*-value
PFOA (log10)	0.05 ( ± 0.18)	0.03 ( ± 0.15)	−0.06 ( ± 0.26)	<0.001
PFOS (log10)	0.19 ( ± 0.19)	−0.03 ( ± 0.23)	−0.29 ( ± 0.26)	<0.001
Age	51 ( ± 9)	58 ( ± 8)	64 ( ± 8)	
BMI	25.1 ( ± 4.3)	26.2 ( ± 4.5)	26.1 ( ± 4.6)	
TC (mmol/L)	5.58 ( ± 1.15)	5.72 ( ± 1.06)	5.78 ( ± 0.95)	<0.001
LDL (mmol/L)	3.51 ( ± 1.05)	3.55 ( ± 0.98)	3.36 ( ± 0.91)	<0.001
HDL (mmol/L)	1.55 ( ± 0.38)	1.69 ( ± 0.48)	1.94 ( ± 0.51)	<0.001
TG (mmol/L)	1.19 ( ± 0.81)	1.07 ( ± 0.58)	1.06 ( ± 0.49)	<0.001
TC:HDL	3.83 ( ± 1.37)	3.64 ( ± 1.17)	3.16 ( ± 0.94)	<0.001
*n* (%)				
Sex				
Female	1135 (99%)	1553 (97%)	1227 (97%)	
Male	10 (0.9%)	48 (3%)	42 (3.3%)	
Zygosity				
MZ	723 (63%)	926 (58%)	729 (57%)	
DZ	422 (37%)	675 (42%)	540 (43%)	

Individuals taking lipid lowering medication have been removed. Differences between the groups were tested using Kruskal–Wallis.

### Twin pair discordance of PFOA and PFOS

A total of 2069 individuals had PFOA and PFOS levels measured, of which 41% were MZ twins, 54% DZ twins, and 5% singletons. There were 971 twin pairs at timepoint 1 and 979 twin pairs at timepoints 2 and 3, (giving 1942 and 1958 individuals in total, with the remaining numbers being singletons within the sample cohort). For the purpose of this analysis, we restricted to the 971 twin pairs with measures across all three timepoints, for a fair comparison (a small number of samples were lost during QC of the metabolite dataset). Twins provide a unique opportunity to consider the within-pair phenotypic discordance, therefore we considered whether PFOA or PFOS levels differed between twin pairs. Twin pair discordance was defined as twins who had PFOA or PFOS levels which were greater than 1 standard deviation from their co-twin.

The total number of discordant twin pairs at each timepoints are displayed in Table 2. We observe at least 25% of twin pairs to be discordant for either PFOA or PFOS at any given timepoint, with the highest discordance observed at timepoint 2 with PFOA (*n* = 320). We observe higher discordance amongst DZ twin pairs than MZ twin pairs, with a higher number of DZ twin pairs discordant both PFOA and PFOS at each timepoint. We further observe 107 twin pairs who remain discordant for PFOA across all three timepoints, and 91 twin pairs who remain discordant for PFOS across all timepoints.

**Table 2:** Number of twin pairs who were discordant for levels of PFOA or PFOS at each timepoint (*n* pairs = 971).

Variable	Timepoint 1	Timepoint 2	Timepoint 3
PFOA N discordant (DZ, MZ)	287 (189, 98)	320 (207, 113)	285 (177, 108)
PFOS N discordant (DZ, MZ)	283 (183, 100)	247 (164, 83)	261 (170, 91)

### PFOA and PFOS display longitudinal associations with lipid traits

**Fig. 2:**
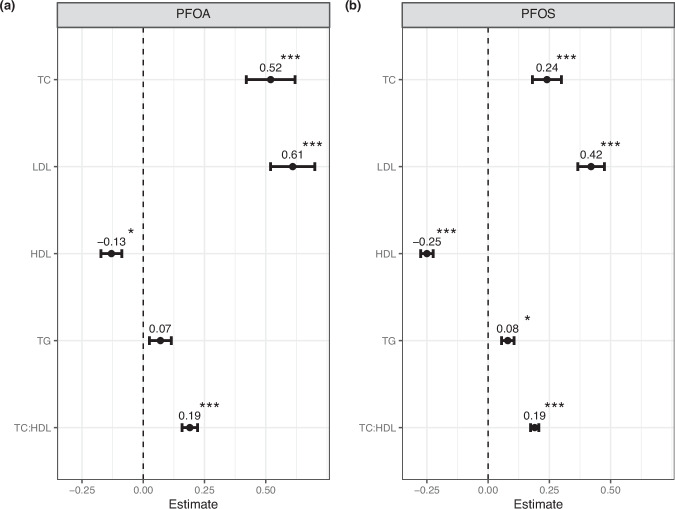
Longitudinal associations between PFAS and lipid phenotypes. Associations with PFOA (**a**) and PFOS (**b**). Plots display coefficient estimates (β) for TC, LDL, HDL, TG and TC:HDL, with standard errors. Significance is denoted by *(*p* < 0.005), **(*p* < 0.001), ***(*p* < 0.0001).

To understand the longitudinal associations of PFOA and PFOS with lipid traits, a longitudinal mixed-effects model was used, with the inclusion of an individual specific random slope to accommodate repeated measures from the same individuals and individual specific trajectories. The outcome of these associations is displayed in Fig. 2 and in Supplementary Table S1. We observed significant associations between PFOS and all lipid traits (Fig. 2b) and between PFOA and all lipid traits, except TG (Fig. 2a). We also observe a consistent direction of effect between PFOA and PFOS with lipid outcomes.

We find TC and LDL to be positively associated with both PFOA and PFOS, with PFOA displaying a larger effect size (TC β = 0.52, *p *= 1.9e−07, LDL β = 0.61, *p *= 1.7e−11). HDL shows significant negative associations with both PFOA (β = −0.12, *p *= 3e−03) and PFOS (β = −0.25, *p *= <2e−16), with a larger effect size evident for PFOS. TG was positively associated with PFOS only (β = 0.08, *p *= 1e−03), however the association with PFOA, whilst not significant did trend in the same direction and displayed a similar effect size. TC:HDL displayed overall positive associations with both PFOA (β = 0.17, *p *= 1.5e−09) and PFOS (β = 0.19, *p *= <2e−16), of similar effect sizes.

### Cross-sectional associations with lipid traits across three timepoints

**Fig. 3:**
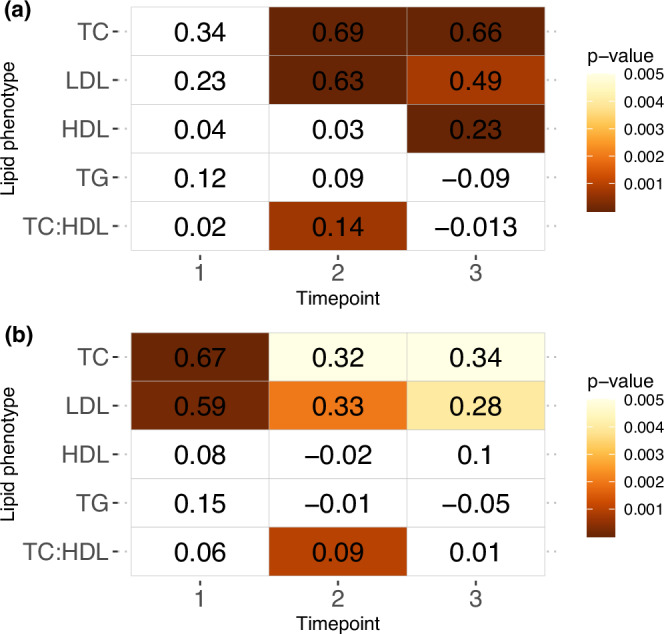
Cross-sectional associations between PFAS and lipid phenotypes across 3 timepoints. Associations with PFOA (**a**) and PFOS (**b**). Heatmap table displays β coefficients and size of *p*-value is indicated by the scale. Non-significant estimates are coloured in white.

In the individual cross-sectional models, we observe consistent positive associations between PFOS with TC and LDL at each of the three timepoints, and time-varying associations to the other lipid phenotypes (Fig. 3, Supplementary Table S2). PFOA also displays significant positive associations with TC and LDL at timepoints 2 and 3. Whilst the association is not significant at timepoint 1, we maintain a consistent positive direct of effect.

There is an overall decrease in the effect size for the associations between PFOS and TC from the first timepoint (β_T1_ = 0.67, *p *= 7.1e−05) to the third timepoint (β_T3_ = 0.34, *p *= 0.005), and between PFOS and LDL across all three timepoints (β_T1_ = 0.59, *p *= 0.0002; β_T2_ = 0.33, *p *= 0.002; β_T3_ = 0.28, *p *= 0.004). PFOA also shows a decrease in the effect size estimates between significant associations at timepoint 2 and 3 for TC (β_T2_ = 0.69, *p *= 1.6e−05, β_T3_ = 0.66, *p *= 2.4e−05) and for LDL (β_T2_ = 0.63, *p *= 1.6e−05, β_T3_ = 0.49, *p *= 0.0008).

Significant positive associations between HDL and PFOA are observed only at timepoint 3 (β = 0.23, *p *= 1.24e−05), with no significant associations between HDL and PFOS. No significant associations are observed with TG and either PFOA or PFOS. Significant positive associations are found between TC:HDL and both PFOA (β = 0.14, *p *= 0.0007) and PFOS (β = 0.09, *p *= 0.001) at timepoint 2.

A sensitivity analysis was conducted, restricting the sample size to retain only individuals who had measures available at all three timepoints (Supplementary Table S3). This was conducted as a cross-sectional analysis to assess the robustness of the findings. We do not observe significant associations at timepoint 2 and do not replicate the significant association between PFOS and LDL at timepoint 3. We still observe all other significant associations at timepoint 1 and timepoint 3, with a positive direction of effect. There is an overall increase in effects sizes for the associations between PFOA with TC and LDL and an overall decrease in the effect sizes between PFOS with TC and LDL.

We further conducted a sensitivity analysis for a mixed-effects model including participants with data across all three timepoints (Supplementary Table S4). We observed no changes to the direction of effect in any model, although the effect size was modestly reduced. The association between TC:HDL and PFOS does not retain significance. Additionally, the associations between PFOS and TC and TG no longer retain statistical significance.

## Discussion

### Main findings

This longitudinal study measured PFOA and PFOS levels over a period of 18 years in 2069 individuals, with 3 repeated measures taken. This study was conducted in a general population cohort of adult twins in the UK. We found PFOA and PFOS levels to be declining over time. Furthermore, we demonstrate discordance of PFOA and PFOS levels between twin pairs, suggesting the importance of exposomic factors in serum PFAS levels.

This study showed the longitudinal associations between PFOA and PFOS and the individual cross-sectional associations with several lipid outcomes in an unselected British population. PFOA and PFOS are positively associated with TC and LDL at both a cross-sectional and longitudinal level. These associations were largely consistent across both longitudinal and cross-sectional findings, including related sensitivity analyses. There is some evidence to suggest PFAS may be associated with HDL levels, although the direction of effect is not consistent across longitudinal and cross-sectional models.

### Previous literature

Previous work has been largely cross-sectional and has reported on positive associations between PFOA and PFOS with TC and LDL [18, 21, 23, 24, 31, 33, 34]. The cross-sectional associations reported here largely agree with the previous literature, finding positive associations between PFOA and PFOS with TC and LDL in individual cross-sectional models. A lower effect size is observed in the associations between PFOS and TC and LDL from the first to last timepoint, in line with the decreasing levels of PFOS over time. We observed a positive association with TC:HDL ratio as reported in one other study [39], and no significant associations with HDL or TG as has been previously noted in some studies [23, 26, 38], other than a positive association with HDL at timepoint 3.

A small number of longitudinal studies have also been conducted to understand the long-term associations between PFAS and lipids. Most of these longitudinal studies continued to support an association between PFOA or PFOS with increased TC or LDL [25, 29, 40–42], although two studies did not find any associations [36, 43]. Two early longitudinal studies in occupationally exposed individuals found statistically significant associations between PFOA and TC [36, 40]. This study also supported associations for increased TC and LDL within this British population cohort. A study of 560 adults, aged 20–60 years at baseline, not taking lipid lowering medication, considered changes in lipids over a 4.4 year period, in a population exposed via contaminated drinking water. PFOA and PFOS serum concentrations had fallen by half, whilst LDL levels did not change substantially. However, those who saw large decrease in PFOA and PFOS levels, also saw a decrease in LDL levels. A 50% decrease in PFOA and PFOS, saw a 3.6% and 5% decrease in LDL levels, respectively. TC and HDL also decreased with halving of PFOA and PFOS, whilst only PFOS was associated with a decrease in triglycerides. This study only uses two timepoints to consider the change in PFOA and PFOS with lipids [41]. A recent comparable longitudinal study further supports this evidence [44]. The study, conducted in the Prospective Investigation of the Vasculature in Uppsala Seniors (PIVUS) cohort in Sweden, took 3 measurements over 10 years, from 864 elderly individuals and found positive associations between the change in PFOA with TC, TG, and HDL-C and between PFOS with HDL-C only [44].

This study also observed positive associations with TG and PFOA and negative associations with HDL. Decreased levels of HDL were significantly associated with both PFOA and PFOS in this study and a positive association was observed between both PFOA and PFOS with TC:HDL ratio, suggestive of an overall negative effect on cholesterol levels, indicating PFOA and PFOS are associated with higher levels of total cholesterol and lower levels of HDL. Whilst one previously published study of women aged 45–56, found an inverse association with TG [29], other studies have reported PFOA or PFOS to be associated with increased TG or HDL [36, 43, 45]. Two studies did not report any significant associations with TG or HDL [40, 41]. Two further longitudinal studies have provided conflicting evidence, with one suggesting a possible increased risk of hypercholesterolaemia and hypertriglyceridemia at baseline [42] and the other finding no evidence of associations with cholesterol, but an inverse relationship with TG [43].

Differences between studies may be due to a combination of factors including age, sex, sample size, control of confounding factors and differing populations and extent of exposure.

Further to this, PFAS concentrations have been shown to differ by age and sex. There has been evidence suggesting that higher mean concentrations of PFAS levels are observed in males [56–58]. A previous study has shown sex is influential in the association between PFAS and lipids [31]. In contrast a recent study found no significant sex-interactions of PFAS and lipid and lipoprotein profiles [59]. Previous studies have also found PFAS are associated with increasing age [57, 58, 60]. Possible explanations for associations with age may be due to longer periods of exposure. Our study is a largely female cohort of older adults, and as such specific age and sex interactions were not considered, however further work is needed to understand the relationship between these factors and the association of PFAS and lipids.

Of the 9 longitudinal studies reported, more than half were in occupationally exposed cohorts or in communities who were exposed via contaminated drinking water, this includes the study conducted in PIVUS, where the Uppsala community has been known to be previously exposed via contaminated drinking water [44, 61]. Of the remaining studies, one only had baseline measures of PFOA and PFOS, and one study was carried out within a diabetic cohort [42]. In contrast to previously published work, this research presents the findings in a healthy general population cohort – i.e. in individuals who are not occupationally exposed or highly exposed through known drinking water contamination. Furthermore, this study utilises 3 repeated measures of both PFAS levels and cholesterol levels, in contrast to most previously published longitudinal studies.

### Mechanisms of action

Whilst the exact mechanisms of action are not fully understood, several proposed mechanisms of lipid perturbations by PFAS have been suggested. One mechanism through which PFAS are thought to perturb lipid homeostasis is through peroxisome proliferator activated receptor alpha (PPARA) activation [62]. PPARA is a nuclear receptor involved in the regulation of hepatic lipid metabolism. Other nuclear receptors and signalling pathways such as PPARG, PXR, CAR and HNF4a, which have been associated with cholesterol and TG homeostasis may also be relevant [47, 62].

A study of mice models found genes of lipid synthesis and degradation were perturbed after PFAS exposure and results in steatosis (fatty liver) [63]. A candidate gene expression study of 290 individuals reported changes to the expression of genes involved cholesterol transport and metabolism, such as *ABCG1* and *NCEH1* [64]. A gene-environment study of 13 single nucleotide polymorphisms (SNPs) across 494 human participants found PPARGC1A and PPARD to be associated with PFOS [65]. Changes in blood lipid levels in humans, following PFAS exposure, may also be mediated by DNA methylation changes [27]. CpG positions annotated to AFF3, CREB5, NRG2, USF2 were found to be associated with PFOA and PFOS in a study of 98 hospitalised patients.

Previous animal studies have reported increased cholesterol after prolonged PFOS exposure and there is some evidence of a dose-response effect [47, 62]. However, in animal models, doses low enough to drive associations in humans, are not seen [62]. Further human studies are required, particularly in populations with chronic low-level exposure, to understand the possible dose response effects, or effects of chronic exposure.

### Strengths and limitations

This study was able to capture longitudinal observations of PFOA and PFOS over a period of 18 years, over three timepoints, in an unselected general population. Associations were considered with several lipids at both a cross-sectional and longitudinal level. This study also has limitations. Missing data and the use of complete case analysis, lead to a reduced sample size. The exclusion of data in this manner may have introduced bias into the study. There were also possible confounders not controlled for in this study, including diet and geosocial factors. We did not have enough data to add kidney function as a possible confounder. Additionally, due to the methods of data collection, there are time differences between timepoints, with a mean difference of 7.1 years between timepoints 1 and 2 and 6.1 years between timepoints 2 and 3. This variability in time may influence the results due to changes in PFOA/PFOS levels and serum lipid levels, and as such, should be considered, when interpreting the results.

Additionally, this study used the Metabolon panel to measure PFOA and PFOS levels. The use of Metabolon gives relative values and not absolute measures. However, despite this we were able to confirm associations with lipid outcomes, which have been previously established using targeted methods to quantify PFOA and PFOS levels. Metabolon has also been used in previous studies to quantify PFOA and PFOS [66, 67], and has been shown to have a strong correlation with targeted methods [50, 51]. In one study, Metabolon measured PFOA/PFOS was compared to targeted measures used by the Centres for Disease Control and Prevention, finding a strong correlation between both measures with a spearman correlation of Rho = 0.76 for both PFOA and PFOS [50]. Another study which specifically addressed the performance of targeted vs non-targeted methods observed a Rho = 0.83 for PFOA and Rho = 0.92 for PFOS, with an agreement of 97–98% between methods [51]. These studies provide confidence that the utilisation of non-targeted methods show a strong correlation with targeted methods.

## Conclusion

This study suggests PFAS may alter lipid homeostasis over time. Further work is needed to better understand the association and impact of PFOA and PFOS on health outcomes. Longitudinal studies provide more information on a given population over time and can assist in deciphering a possible causal relationship. Given that levels of PFOA and PFOS are decreasing, longitudinal studies could help provide a better understanding of the relationship between these chemicals and long-term health outcomes. Given the long-term associations of PFAS on lipids suggested within this study, and the widespread potential for exposure of these chemicals, future work should focus on understanding risks of low level, chronic exposure.

## Supplementary information

Supplementary information

## Data Availability

Data is available via application to the TwinsUK Resource Executive Committee. Information on data access and how to apply is available at https://twinsuk.ac.uk/researchers/access-data-and-samples/request-access/.
