# Correction: Longitudinal association of perfluorooctanoic acid (PFOA) and perfluorooctanesulfonic acid (PFOS) exposure with lipid traits, in a healthy unselected population

**DOI:** 10.1038/s41370-025-00792-0

**Published:** 2025-07-18

**Authors:** Yasrab N. Raza, Julia S. El-Sayed Moustafa, Xinyuan Zhang, Dongmeng Wang, Max Tomlinson, Mario Falchi, Cristina Menni, Ruth C. E. Bowyer, Claire J. Steves, Kerrin S. Small

**Affiliations:** 1 Department of Twin Research and Genetic Epidemiology, King’s College London, London, UK; 2 Department of Pathophysiology and Transplantation, Università Degli Studi di Milano, Via Francesco Sforza, 35, 20122 Milan, Italy; 3 Fondazione IRCCS Cà Granda Ospedale Maggiore Policlinico, Angelo Bianchi Bonomi Hemophilia and Thrombosis Center, 20122 Milan, Italy

Correction to: *Journal of Exposure Science & Environmental Epidemiology* 10.1038/s41370-025-00773-3, published online 24 April 2025

In the sentence beginning ‘We observe higher discordance amongst...’ in this article and in table 2, column ‘Variable’, the term ‘MZ’ should have read ‘DZ’ and vice versa. The corrected table is given below.

The original article has been corrected.

Table 2 Number of twin pairs who were discordant for levels of PFOA or PFOS at each timepoint (*n* pairs = 971).

**Table:** 

Variable	Timepoint 1	Timepoint 2	Timepoint 3
PFOA N discordant (DZ, MZ)	287 (189, 98)	320 (207, 113)	285 (177, 108)
PFOS N discordant (DZ, MZ)	283 (183, 100)	247 (164, 83)	261 (170, 91)

