# Supplementary material for: Longitudinal association of Perfluorooctanoic Acid (PFOA) and Perfluorooctanesulphonic Acid (PFOS) exposure with lipid traits, in a healthy unselected population
Source: J Expo Sci Environ Epidemiol. Author manuscript; Available in PMC 2025 Jun 9. (PMC7617748; doi:10.1038/s41370-025-00773-3)
Supplement: Supplementary file 1 [file EMS204499-supplement-Supplementary_Material.pdf]

Supplementary Material

Supplementary Methods

Sample Preparation: Samples were kept at -80°C until processed. Samples were extracted with methanol and underwent non-targeted MS analysis using UPLC-MS/MS, as described [1,2]. 100ul of each serum sample was prepared using the automated MicroLab STAR® system from Hamilton Company. For quality control, several recovery standards were added prior to initial extraction. Proteins were precipitated uing methanol, followed by vigorous shaking for 2 minutes (Glen Mills GenoGrinder 2000) and centrifugation. Extracts were divided into five fractions; two for analysis by two separate reverse phase (RP)/UPLC-MS/MS methods with position ion mode electrospray ionization (ESI), one for RP/UPLC-MS/MS and one for HILIC/UPLC-MS/MS, both with negative ion mode ESI, and one fraction reserved for backup. To remove the organic solvent, samples were briefly placed on a TurboVap® (Zymark). The sample extracts were stored overnight under nitrogen before being prepared for analysis.

Several types of controls were analysed alongside experimental samples. A pool of well-characterised human plasma purchased from bioreclamation served as a technical replicate throughout the datasets. Extracted water samples acted as process blanks, and a carefully selected cocktail of QC standards – chosen to avoid interference with the measurement of endogenous compounds – were spiked into every analysed compound. This allowed instrument performance monitoring and aided in chromatographic alignment. Instrument variability was determined by calculating the median relative standard deviation (RSD) for the internal standards that were added to each sample prior to injection into the mass spectrometers. Overall process variability was determined by calculating the median RSD for all endogenous metabolites present in 100% of the pooled matrix samples. Experimental samples were randomized across the platform run with QC samples spaced evenly among the injections.

Ultrahigh Performance Liquid Chromatography–Tandem Mass Spectroscopy (UPLC–MS/MS): Sample extracts were dried then reconstituted in solvents compatible to each of the four methods. All methods utilized a Waters ACQUITY ultra-performance liquid chromatography (UPLC) and a Thermo Scientific Q-Exactive high resolution/accurate mass spectrometer interfaced with a heated electrospray ionization (HESI-II) source and Orbitrap mass analyzer operated at 35,000 mass resolution. Each reconstitution solvent contained a series of standards at fixed concentrations to ensure injection and chromatographic consistency. One aliquot was analyzed using acidic positive ion conditions,

chromatographically optimized for more hydrophilic compounds. In this method, the extract was gradient eluted from a C18 column (Waters UPLC BEH C18-2.1x100 mm, 1.7 µm) using water and methanol, containing 0.05% perfluoropentanoic acid (PFPA) and 0.1% formic acid (FA). Another aliquot was also analyzed using acidic positive ion conditions; however, it was chromatographically optimized for more hydrophobic compounds. In this method, the extract was gradient eluted from the same afore mentioned C18 column using methanol, acetonitrile, water, 0.05% PFPA and 0.01% FA and was operated at an overall higher organic content. A third aliquot was analyzed using basic negative ion optimized conditions using a separate dedicated C18 column. The basic extracts were gradient eluted from the column using methanol and water, however with 6.5mM Ammonium Bicarbonate at pH 8. The fourth aliquot was analyzed via negative ionization following elution from a HILIC column (Waters UPLC BEH Amide 2.1x150 mm, 1.7 µm) using a gradient consisting of water and acetonitrile with 10mM Ammonium Formate, pH 10.8. The MS analysis alternated between MS and data-dependent MSn scans using dynamic exclusion. The scan range methods covered 70-1000 m/z.

Data Quality: Technical replicates were analysed to assess matrix effects and distinguish between biological and process variability. Serum technical replicates were created by pooling a small aliquot from each experimental sample, which were then injected periodically throughout the platform run. Variability among consistently detected biochemicals in the technical replicates was then used to calculate the RSD for endogenous metabolites. The RSD for PFOA and PFOS were 15.9079 and 12.85181, respectively.

Compound Identification & Metabolite Quantification: Raw data were extracted, peak-identified and QC processed using Metabolon's software. Metabolites were identified by automated comparison of the ion features in the experimental samples to a reference library of chemical standard entries that included retention time, molecular weight (m/z), preferred adducts, and in-source fragments as well as associated MS spectra and curated by visual inspection for quality control using software developed at Metabolon.

Identification of known chemical entities was based on comparison to metabolomic library entries of purified standards. Commercially available purified standard compounds have been acquired for determination of their detectable characteristics. Additional mass spectral entries have been created for structurally unnamed biochemicals, which have been identified by virtue of their recurrent nature (both chromatographic and mass spectral). Peaks were quantified using area-under-the-curve. Raw area counts for each metabolite in each sample were normalized to correct for variation resulting from instrument inter-day tuning differences

by the median value for each run-day, therefore, setting the medians to 1.0 for each run. Missing data was imputed using nonparametric missing value imputation using random forest.

1. Bridgewater BR, E. A. High Resolution Mass Spectrometry Improves Data Quantity and Quality as Compared to Unit Mass Resolution Mass Spectrometry in High-Throughput Profiling Metabolomics. *Journal of Postgenomics Drug & Biomarker Development* **04**, (2014).
2. Hysi, P. G. *et al.* Metabolome Genome-Wide Association Study Identifies 74 Novel Genomic Regions Influencing Plasma Metabolites Levels. *Metabolites* **12**, (2022).

Table S1: Results of longitudinal linear regression output with lipid phenotypes

	PFOA		PFOS	
**Phenotype**	**β (se)**	**p-value**	**β (se)**	**p-value**
TC (n=4010)	0.52 (0.10)	1.9e-07	0.24 (0.06)	3.8e-05
LDL (n=3929)	0.61 (0.09)	1.8e-11	0.42 (0.05)	1.6e-14
HDL (n=4011)	-0.13 (0.04)	3e-03	-0.25 (0.03)	< 2e-16
TG (n=3934)	0.07 (0.04)	0.1	0.08 (0.03)	1e-03
TC:HDL (n=4007)	0.19 (0.03)	4.8e-09	0.19 (0.02)	< 2e-16

Table S2: Results of cross-sectional linear regression for lipid phenotypes

PFAS	Phenotype	Timepoint 1		Timepoint 2		Timepoint 3	
		**β (se)**	**p-value**	**β (se)**	**p-value**	**β (se)**	**p-value**
PFOA	TC	0.34 (0.17)	0.046	0.69 (0.16)	1.6e-05	0.66 (0.16)	2.4e-05
	LDL	0.23 (0.16)	0.14	0.63 (0.15)	1.6e-05	0.49 (0.15)	0.0008
	HDL	0.04 (0.06)	0.49	0.03 (0.06)	0.59	0.23 (0.06)	1.2e-05
	TG	0.12 (0.08)	0.13	0.09 (0.07)	0.14	-0.09 (0.06)	0.13
	TC:HDL	0.02 (0.05)	0.65	0.14 (0.04)	0.0007	-0.013 (0.04)	0.73
PFOS	TC	0.67 (0.17)	7.1e-05	0.32 (0.11)	0.005	0.34 (0.1)	0.005
	LDL	0.59 (0.16)	0.0002	0.33 (0.11)	0.002	0.28 (0.1)	0.004
	HDL	0.08 (0.06)	0.16	-0.02 (0.04)	0.73	0.1 (0.04)	0.02
	TG	0.15 (0.08)	0.05	-0.01 (0.05)	0.914	-0.05 (0.38)	0.23
	TC:HDL	0.06 (0.05)	0.27	0.09 (0.03)	0.001	0.01 (0.03)	0.6

Table S3: Results of cross-sectional linear regression for lipid phenotypes (n=622)

PFAS	Phenotype	Timepoint 1		Timepoint 2		Timepoint 3	
		β (se)	p-value	β (se)	p-value	β (se)	p-value
PFOA	TC	0.42 (0.23)	0.06	0.49 (0.25)	0.05	0.95 (0.23)	3.35e-11
	LDL	0.44 (0.2)	0.03	0.31 (0.22)	0.16	0.62 (0.22)	0.005
	HDL	0.004 (0.08)	0.95	0.17 (0.1)	0.1	0.39 (0.1)	0.0001
	TG	0.004 (0.1)	0.97	-0.01 (0.1)	0.9	-0.03 (0.09)	0.7
	TC:HDL	0.03 (0.08)	0.68	-0.03 (0.06)	0.66	-0.03 (0.06)	0.6
PFOS	TC	0.6 (0.21)	7.1e-05	0.19 (0.16)	0.24	0.45 (0.15)	0.003
	LDL	0.59 (0.16)	0.004	0.21 (0.14)	0.14	0.36 (0.14)	0.01
	HDL	0.12 (0.07)	0.08	0.02 (0.07)	0.73	0.15 (0.07)	0.03
	TG	0.05 (0.1)	0.6	-0.04 (0.07)	0.52	-0.07 (0.06)	0.25
	TC:HDL	-0.007 (0.07)	0.9	0.04 (0.04)	0.27	0.008 (0.04)	0.8

Table S4: Results of longitudinal linear regression output with lipid phenotypes (n=622)

	PFOA		PFOS	
Phenotype	β (se)	p-value	β (se)	p-value
TC	0.43 (0.15)	4.2e-03	0.13 (0.09)	0.14
LDL	0.57 (0.13)	1.4e-05	0.34 (0.08)	2.0e-05
HDL	-0.07 (0.06)	3e-03	-0.19 (0.04)	1.3e-07
TG	0.01 (0.06)	0.9	0.007 (0.04)	0.8
TC:HDL	0.13 (0.05)	0.02	0.14 (0.03)	3.91e-08

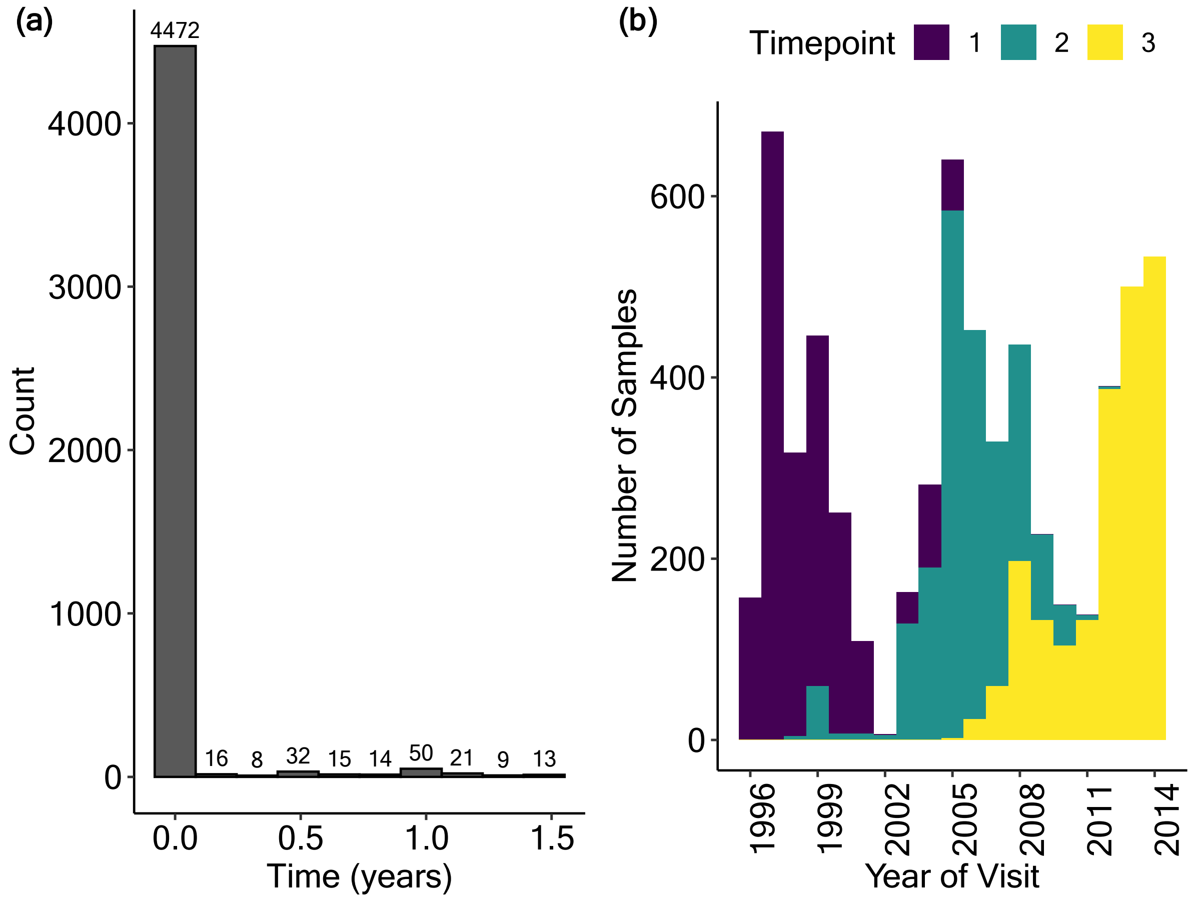

Fig. S1: (a) time difference in years between PFAS sample collection date and lipid sample collection date (b) year of collection of (PFAS) Metabolon samples stratified by timepoint.
